# A Systematic Review of Research and Governance in Child and Adolescent Mental Health in Africa

**DOI:** 10.1111/tmi.70034

**Published:** 2025-09-16

**Authors:** Margaret Isioma Ojeahere, Emelia Pasternak‐Albert, Mercury Shitindo, Lily Kpobi, Christopher Goson Piwuna, Tolulope Olumide Afolaranmi, Mariana Pinto da Costa

**Affiliations:** ^1^ Department of Psychiatry Jos University Teaching Hospital Plateau State Nigeria; ^2^ Institute of Psychiatry, Psychology & Neuroscience King's College London London UK; ^3^ Africa Bioethics Network Nairobi Kenya; ^4^ Regional Institute for Population Studies University of Ghana Legon Accra Ghana; ^5^ Department of Psychiatry, College of Health Sciences University of Jos Plateau State Nigeria; ^6^ Department of Community Medicine, College of Health Sciences University of Jos Plateau State Nigeria; ^7^ Institute of Biomedical Sciences Abel Salazar, University of Porto Porto Portugal

**Keywords:** ethics, mental health, research design

## Abstract

**Background:**

Children and adolescents with mental health conditions represent a uniquely vulnerable population, particularly in Africa where mental health systems are under‐resourced and understudied. Conducting research with this group raises complex ethical questions that require robust legislative and ethical oversight.

**Methods:**

This systematic review included searches from Medline, Embase, PsycInfo, Global Health and grey literature, conducted without any time restrictions up to 10 September 2023. Publications included focused on individuals aged 0–17. All study designs were included if they addressed governance and regulations in child and adolescent mental health research in Africa. Excluded publications did not report findings specific to children, to Africa, to governance or regulations in mental health research, or had no available full text. Articles were critically appraised using JBI checklists and data was extracted into Excel. Articles were narratively synthesised using frequencies for dates of regulations and ages of consent and coded in NVivo for attitudes toward regulation. The study protocol is available at PROSPERO (CRD42023464864).

**Results:**

This review identified 14 articles from nine countries across Africa. Most regulations were over a decade old, with the most recent from 2017. The publications covered five themes: concerns toward unfavourable existing legislation, concerns about risks of undertaking research on a clinical frontline, a call to action regarding the dearth of African literature in this field, specific recommendations for future research and suggested new research directions.

**Conclusions:**

This study highlights the need for improved research governance and legislation to protect children and adolescents in mental health research in Africa. Overall, most African countries place a low priority on child and adolescent mental health research.

**Abbreviations**
CIOMS: Council of International Organizations of Medical Sciences
WHO: World Health Organization

## 1 Introduction

Children and adolescents are vulnerable to mental health conditions. Globally, it is estimated that 14% of individuals aged 10–19 years suffer from a mental health condition, accounting for 13% of the total disease burden in this age group [1]. Recent studies in sub‐Saharan Africa suggest that approximately 14.3% of children and adolescents experience significant psychological difficulties, while 9.5% meet the criteria for a mental health condition [2]. However, these figures may underestimate the true extent of the problem due to the scarcity and geographical imbalance of research on children and adolescents in Africa.

To accurately assess the magnitude of mental health conditions in this demographic in Africa, there is an urgent need for more comprehensive, high‐quality and ethically sound research. A recent systematic review highlighted an increase in mental health‐focused epidemiological studies in sub‐Saharan Africa, providing a more representative picture of mental health needs among children and adolescents in the region [3].

Despite the critical importance of ethical considerations and legislative frameworks guiding and safeguarding the participation of children and adolescents in mental health research, such frameworks are often unclear in the African context. While global guidelines such as the *Nuremberg Code*, the *Helsinki Declaration* and the *Belmont Report* protect human research participants, these documents have historically focused on adults [4, 5, 6, 7]. More recent normative documents, such as the 2021 edition of the *International Compilation of Human Research Standards* [8] are predominantly centred on adults. The recognition of children and adolescents as unique research participants emerged, with the 1993 revision of the *Helsinki Declaration* and the *International Ethical Guidelines for Biomedical Research Involving Humans* by the Council of International Organizations of Medical Sciences (CIOMS) in collaboration with the World Health Organization (WHO) [9]. The United Nations *Convention on the Rights of the Child* also emphasises the importance of respecting children's autonomy and ensuring their protection [10, 11].

Despite these frameworks, children and adolescents under 18 are often underrepresented in research due to ethical and structural barriers [12]. In many African countries, individuals under 18 are considered minors, requiring parental consent, which complicates their participation in research. This raises ethical dilemmas concerning autonomy, beneficence, nonmaleficence and justice, with issues such as assent, informed consent, confidentiality and researcher competence. Children and adolescents with mental health conditions may not fully understand the research scope or provide informed consent, potentially conflicting with the *Nuremberg Code*'s principle of voluntary and informed participation. Furthermore, the lack of clear regulations for mental health research involving children and adolescents in Africa may predispose this vulnerable population to potential exploitation as research participants and hampers the advancement of research in this field.

In many cases, children and adolescents with mental health conditions may not be capable of giving assent or fully understanding the scope of the research, contradicting the *Belmont Report*'s principle that participants must freely consent to participate, understanding the nature of the research and the potential risks and benefits [7]. As advancements in biotechnology and calls for social justice grow, the likelihood of ethical dilemmas in research involving children and adolescents will increase, potentially leading to exploitation in mental health research.

Thus, robust governance in mental health research is crucial to prevent unethical practices, enhance research standards and ensure the upholding of integrity and ethics. Effective governance fosters ethical research, leading to interventions, programmes and policies that benefit children and adolescents [13]. However, in parts of Africa, the state of governance and ethics concerning research involving children and adolescents with mental health conditions remains ambiguous. Currently, only a few African countries have established laws to regulate research in this demographic, and ethical issues related to this group are still poorly understood [14].

This systematic review aims to address these gaps by exploring the following research questions: (1) What regulations guide mental health research involving children and adolescents in Africa? (2) What ethical guidelines and informed consent procedures are in place for mental health research with children and adolescents in Africa?

## 2 Methods

### 2.1 Search Strategy and Selection Criteria

This systematic review was conducted in accordance with PRISMA guidelines [15]. The study protocol is available at PROSPERO (CRD42023464864). We searched MEDLINE, Embase, PsycINFO and Global Health with no language or time restrictions. The Embase search had an age limitation for 17 years and under, according to the predefined categories as follows: (embryo or infant or child or preschool child < 1–> 6 years or school child < 7–> 12 years or adolescent < 13–> 17 years). Subject headings, MeSH terms and search terms pertaining to children and adolescents (e.g., ‘child’ and ‘adolescen*’), governance or consent (e.g., ‘legislation’ and ‘assent’), mental health (e.g., ‘anxiety’ and ‘depression’) and Africa (e.g., ‘Kenya’ and ‘Nigeria’) were combined using Boolean operators. The full search strategy is provided in the Supporting Information: Data S1. Articles included were published between each database's inception and 10 September 2023.

Publications were included if they focused on individuals aged 17 years or younger. All study designs were included. Editorials, letters, perspectives, commentaries, book chapters, dissertations and conference proceedings were included if they addressed governance and regulations in child and adolescent mental health research in Africa.

Publications were excluded if they included both children and participants older than 17 years but did not report findings specific to children, or if they covered both African and non‐African countries but did not provide data specific to Africa, or if they did not report on governance or regulations in mental health research, or if the full text was unavailable.

Grey literature was sourced using Google Scholar search engines such as African Journals Online and the Westlaw International law database.

The search results were exported to EndNote, where duplicates were removed. The remaining publications were screened by two authors for title and abstract eligibility. Full‐text screening was conducted independently by two authors, with discrepancies resolved through discussion or by consulting a third reviewer.

### 2.2 Data Analysis

A data extraction template was developed in Excel, listing items such as study design and African nation studied. Two authors independently extracted data from each publication, and any discrepancies were resolved through discussion with a third author.

Risk of bias was assessed using the Joanna Briggs Critical Appraisal Tools for case series [16], qualitative research [17], text and opinion [18] and systematic reviews [19]. Results are presented in the Supporting Information: Data S1.

Thematic analysis of the full‐text document was conducted using NVivo. This analysis explored attitudes toward regulation, identified reasons for the lack of literature and compared regulations across African countries. Ethical issues in mental health research and governance strategies were also evaluated. Themes and subthemes were generated.

A descriptive quantitative analysis was also performed, representing frequencies in data extraction. Heterogeneity across the publications was assessed by categorising data by African nation.

## 3 Results

**FIGURE 1:**
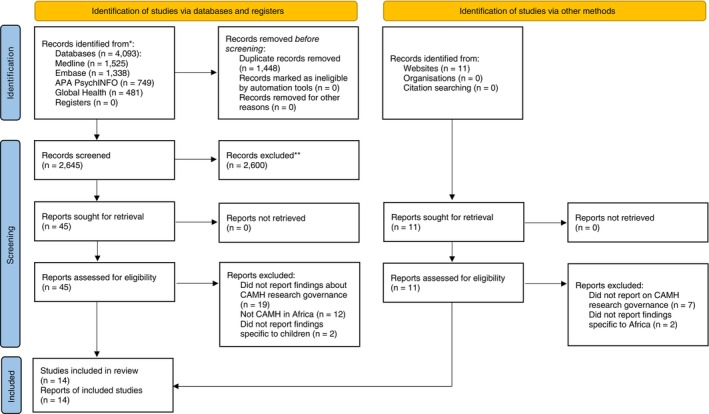
Study selection.

Our initial database search identified 4093 articles. After removing duplicates, 2645 remained and were screened based on titles and abstracts. We conducted full‐text screenings for 45 publications and 11 additional ones from grey literature searches, resulting in 14 publications being included in the synthesis. Figure 1 outlines the study selection process, while excluded publications are listed in the Supporting Information: Data S1. Most publications were excluded because they did not address the regulation of child and adolescent mental health research in Africa.

The included publications covered nine African countries: South Africa (*n* = 9, 64%), Kenya (*n* = 3), Nigeria (*n* = 2), Uganda (*n* = 2), Zambia (*n* = 2), Ethiopia (*n* = 1), Ghana (*n* = 1), Rwanda (*n* = 1) and Tunisia (*n* = 1). One paper studied six African countries [20], but most (*n* = 11, 79%) focused on just one country [21]. Included publications varied in origin and design, including two book chapters [22, 23], a legal document [24], and other formats such as letters to the editor, scoping reviews, situational analyses and commentaries. Most (*n* = 10, 71%) of the publications discussed child and adolescent mental health broadly, while one focused on autism spectrum disorder [25], two on HIV‐related mental health [26, 27], and one on suicide risk [28].

Eight publications (57%) referenced existing research legislation or governance practices. Most regulations were over a decade old, with seven (50%) from the 2010s, six (43%) from the 2000s, two from the 1990s and one referred to regulations from the 1970s (the Belmont report 1979) and 1960s (Declaration of Helsinki 1964) [28]. Two articles focused on Section 71 of South Africa's National Health Act, enacted in 2012 [26, 29]. The most recent regulation discussed was Uganda's 2017 child and adolescent mental health policy [20].

There was no clear consensus on the age group covered by the term ‘child and adolescent mental health services’, with age ranges from 0 to 24 years. Ethical concerns were linked to the age of consent, which were set at 18 years in all countries that explicitly mentioned them. Five publications (36%) did not specify the age of the study population. One article highlighted that historically, South Africa defined legally ‘child’ (up to 18 years) and ‘minor’ (up to 21 years) [29].

Ten (71%) publications did not state an age of consent for research participation. One article specifically commented on how South Africa missed an opportunity to regulate this, as their Children's Bill set an age of consent for medical treatment, but not for research participation [30]. Another article discussed how the age of consent for participating in therapeutic research in South Africa was 14 years before the 2012 National Health Act [29].

Some publications (*n* = 3, 21%) noted that in Rwanda, assent and consent were used interchangeably [27] while South Africa did not use assent in its legal system [24, 29].

Of the 10 articles (71%) that discussed child and adolescent mental health policies, child and adolescent mental health research was viewed either as a low priority (*n* = 7, 70%) or not a priority at all (*n* = 3, 30%). One article noted that Nigeria's Ministry of Health policy goals mentioned mental health but not children's mental health [27].

The themes emerging from the thematic analysis, detailed in the Supporting Information: Data S1, revealed several concerns including that existing legislation was viewed as unfavourable to researchers and ethics committees. There was a call for action to address barriers, risks and challenges related to undertaking research with this vulnerable population, as well as specific recommendations for future research directions.

Concerns about unfavourable existing legislation included criticism of the requirement for research to be therapeutic (*n* = 4), discrepancies between legislation and the proceedings of ethics committees (*n* = 3), conflicts between children's rights to privacy and participation in research (*n* = 1), and burdensome bureaucratic processes (*n* = 1).

Risks to researchers included working on the clinical frontline for this population (*n* = 5), that vulnerable children without parents who can consent to their participation are not considered when devising research legislation (*n* = 3), and challenges conducting school‐based research which can be subjected to laws around suicidality (*n* = 1).

The call to action focused on addressing the dearth of literature on this topic (*n* = 18) and a lack of recognition of existing research (*n* = 2). This lack of acknowledgement was discussed across seven of the reports, with eight reports detailing ways the research landscape could change.

Specific recommendations included fostering intersector work and co‐produced research (*n* = 8), developing new information governance methodologies (*n* = 4), increasing funding for child and adolescent mental health research (*n* = 3), and legislation supporting emerging researchers (*n* = 3).

New directions for research included epidemiological research (*n* = 2), school‐based research (*n* = 2), neurodivergence‐informed research (*n* = 1) and research into consent and capacity‐gaining processes in this population (*n* = 1).

**FIGURE 2:**
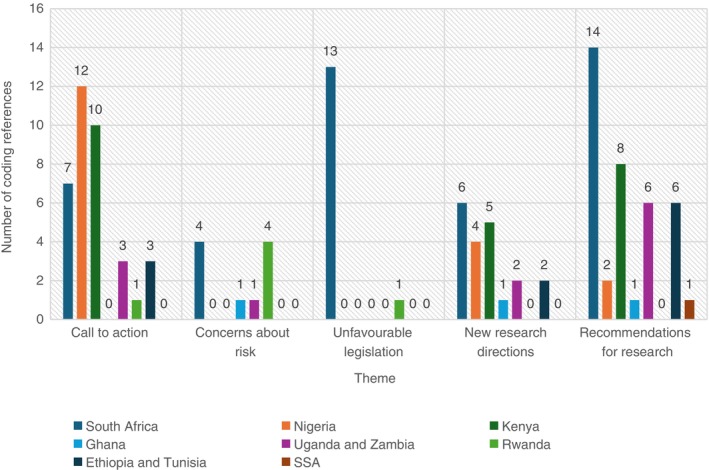
Frequency of themes coded stratified by African country. Of the 14 publications, five (36%) collected primary data, both qualitative (*n* = 4) [20, 26, 27, 31] and quantitative (*n* = 2) [31, 32], and the rest (*n* = 9, 64%) were text/opinion including narrative and scoping reviews. Themes were only extracted if they were supported by at least four articles to ensure that they were substantiated.

Figure 2 presents the distribution of coding references, stratified by the African countries. The unfavourable legislation referenced from South Africa was predominantly Section 71 of the National Health Act 2012, which mandated parental consent for child participation in research.

## 4 Discussion

This systematic review examines ethical considerations and regulations governing research involving children and adolescents with mental health conditions in Africa. While considerable work has been done globally, research from Africa remains scarce, despite its importance in shaping legislation and advancing science [13, 23, 29]. The publications reviewed examined policies and legislation guiding mental health research involving children and adolescents across all the regions of Africa [20, 21, 22, 23, 24, 25, 26, 27, 28, 29, 30, 31, 32, 33]. South Africa accounted for the majority (*n* = 9) of publications [20, 21, 24, 26, 29, 30, 31, 32, 33] highlighting the need for more research from other regions of Africa. Child and adolescent mental health research is often not prioritised across the continent [20, 22, 23, 25, 26, 28, 30, 31, 32, 33] leaving significant gaps in addressing their mental health needs.

Five key themes emerged regarding the ethical and legislative challenges in conducting research on children and adolescents with mental health conditions: a call to action, existing legislations as barriers, risks of conducting research, recommendations for future research and new directions for research.

Our findings revealed outdated legislation on child and adolescent mental health research, with most laws enacted over a decade old [26, 28, 29, 32]. This aligns with previous studies reporting weak health research governance [13, 34]. The need for periodic reviews of policies and legislation to reflect contemporary challenges is evident. The most recent report was Uganda's 2017 Child and Adolescent Mental Health Policy, aimed at promoting mental health and preventing mental, neurological and substance use disorders among children and adolescents [20]. A World Health Organization (WHO) report showed that many African countries have limited mental health policies or plans, and only a few have updated their policies to address current challenges [35]. Nigeria's Mental Health Act of 2021, signed into law in 2023, notably lacks specific regulations for research involving children and adolescents [36]. Many African countries still lack robust health research governance structures, despite the endorsement of a health strategy in August 2022 by African health ministers to ensure the development and implementation of policies and legislation promoting scientific progress in mental health care by 2030 [37, 38]. Our findings indicate that the majority of African countries do not have national health research systems to set standards for child and adolescent psychiatry research, including research capacity and adequate information governance guidance. The absence of legislation and poor implementation of existing policies can perpetuate unethical research practices, potentially exploiting children and adolescents as participants and exacerbating their mental health challenges [13, 14, 39].

Ethical considerations involving children and adolescents are receiving growing attention, particularly regarding informed consent, assent, confidentiality and power dynamics [12, 14, 39]. Upholding ethical principles is essential to protect children and adolescents in mental health research. Without these safeguards, children and adolescents with mental health conditions may face adverse experiences participating in research, compounded by their perceived lack of competency and autonomy, as well as the complexities of mental health conditions [39]. Our review found a lack of consensus regarding the age of children and adolescents, the age of consent, and assent in mental health research in Africa [24, 27, 29, 30]. This is consistent with previous findings from both African and international research ethics guidelines, which provide limited guidance on assent and parental consent, with the notable exceptions of the CIOMS and ICH‐GCP guidelines [14, 40]. Assent, a crucial ethical consideration in paediatric research, was largely overlooked in several of the publications and was sometimes used interchangeably with consent [24, 27, 28]. This suggests that researchers and policy makers may not fully appreciate the importance of these ethical principles, emphasising the need for clearer definitions. The vulnerability of minors demands additional protection from clinicians, researchers and policymakers, beyond what is provided to competent adult participants.

A significant gap identified in this review is the scarcity of information on this topic in Africa, although empirical evidence suggests that research on children and adolescents is being conducted in Africa [41, 42], barriers to dissemination limit its visibility [42, 43, 44].

This systematic review has several strengths, including its rigorous methodology and the inclusion of diverse publications, most of which were rated as moderate to high quality. However, limitations include the exclusion of non‐English‐language articles and the restriction to participants under 18, who are still considered minors in much of Africa.

This review highlights the need for improved research governance and legislation to protect children and adolescents in mental health research. Our findings emphasise the importance of ethical integrity and the need to harmonise consent and assent requirements for research involving children and adolescents with mental health conditions in Africa. Future research should focus on assessing the capacity for consent and assent in mental health research, particularly for children without parents who can consent for them.

Collaboration from stakeholders such as children and adolescents with lived experiences and their caregivers is essential, as is intersectoral cooperation between clinicians, school staff, researchers and policymakers. Future research might also explore how laws surrounding suicidality in specific African countries affect school‐based mental health research.

Beyond conducting research in Africa, publishing findings is crucial for driving scientific progress and improving mental health care. Policymakers should prioritise funding and support for research in this field.

## Disclosure

The funder had no role in study design, data collection, analysis, interpretation or writing of the report.

## Conflicts of Interest

The authors declare no conflicts of interest.

## Supporting information


**Data S1:** Supporting Information.

## Data Availability

The study protocol is available to access from the PROSPERO database. CRD42023464864. URL: https://www.crd.york.ac.uk/prospero/display_record.php?ID=CRD42023464864.
