# Supplementary material for: A Systematic Review of Research and Governance in Child and Adolescent Mental Health in Africa
Source: Trop Med Int Health. 2025 Sep 16;30(11):1254–60. doi: 10.1111/tmi.70034 (PMC12588803; doi:10.1111/tmi.70034)
Supplement: Supplementary file 1 — Data S1: Supporting Information. [file TMI-30-1254-s001.docx]

**Supplementary Material**

# **Appendix 1. PRISMA Checklist**^1^

| **Section/topic** | **#** | **Checklist item** | **Reported on page #** |
| --- | --- | --- | --- |
| **TITLE** |  |  |  |
| Title | 1 | Identify the report as a systematic review, meta-analysis, or both. | 1 |
| **ABSTRACT** |  |  |  |
| Structured summary | 2 | Provide a structured summary including, as applicable: background; objectives; data sources; study eligibility criteria, participants, and interventions; study appraisal and synthesis methods; results; limitations; conclusions and implications of key findings; systematic review registration number. | 2 |
| **INTRODUCTION** |  |  |  |
| Rationale | 3 | Describe the rationale for the review in the context of what is already known. | 3-4 |
| Objectives | 4 | Provide an explicit statement of questions being addressed with reference to participants, interventions, comparisons, outcomes, and study design (PICOS). | 4 |
| **METHODS** |  |  |  |
| Protocol and registration | 5 | Indicate if a review protocol exists, if and where it can be accessed (e.g., Web address), and, if available, provide registration information including registration number. | 4 |
| Eligibility criteria | 6 | Specify study characteristics (e.g., PICOS, length of follow-up) and report characteristics (e.g., years considered, language, publication status) used as criteria for eligibility, giving rationale. | 4 & appendix |
| Information sources | 7 | Describe all information sources (e.g., databases with dates of coverage, contact with study authors to identify additional studies) in the search and date last searched. | 4 & appendix |
| Search | 8 | Present full electronic search strategy for at least one database, including any limits used, such that it could be repeated. | Appendix |
| Study selection | 9 | State the process for selecting studies (i.e., screening, eligibility, included in systematic review, and, if applicable, included in the meta-analysis). | 4 & appendix |
| Data collection process | 10 | Describe method of data extraction from reports (e.g., piloted forms, independently, in duplicate) and any processes for obtaining and confirming data from investigators. | 4 & appendix |
| Data items | 11 | List and define all variables for which data were sought (e.g., PICOS, funding sources) and any assumptions and simplifications made. | 4 & appendix |
| Risk of bias in individual studies | 12 | Describe methods used for assessing risk of bias of individual studies (including specification of whether this was done at the study or outcome level), and how this information is to be used in any data synthesis. | 4 & appendix |
| Summary measures | 13 | State the principal summary measures (e.g., risk ratio, difference in means). | N/A |
| Synthesis of results | 14 | Describe the methods of handling data and combining results of studies, if done, including measures of consistency (e.g., I^2^) for each meta-analysis. | N/A |

# **Appendix 2. Search syntax**

| **Medline** | | |
| --- | --- | --- |
| # | **Searches** | **Results** |
| 1 | Exp child/ | 2160742 |
| 2 | Exp adolescent/ | 2220823 |
| 3 | Exp Pediatrics/ | 63124 |
| 4 | Infant*·ti,ab· | 461132 |
| 5 | Toddler*·ti,ab· | 14418 |
| 6 | Child·ti,ab· | 396024 |
| 7 | Children·ti,ab· | 1217252 |
| 8 | Teen*·ti,ab· | 35337 |
| 9 | Youth*·ti,ab· | 97977 |
| 10 | Youngster*·ti,ab· | 2779 |
| 11 | Adolescen*·ti,ab· | 350469 |
| 12 | Pre?school*·ti,ab· | 34874 |
| 13 | Kindergarten*·ti,ab· | 8228 |
| 14 | School*·ti,ab· | 344732 |
| 15 | Juvenile*·ti,ab· | 94331 |
| 16 | P?ediatric*·ti,ab· | 448321 |
| 17 | (young adj2 (adult* or person* or individual* or people* or population* or man or men or wom#n)) ·ti,ab· | 238023 |
| 18 | (secondary adj2 (school* or education)) ·ti,ab· | 18641 |
| 19 | Mental Health/ | 62431 |
| 20 | Exp Mental Disorders/ | 1440945 |
| 21 | Exp Psychiatry/ | 110778 |
| 22 | Exp Child Psychiatry/ | 5806 |
| 23 | *Mental Health Services/ | 30628 |
| 24 | *Intellectual Disability/ | 43386 |
| 25 | *Depression/di, ep [Diagnosis, Epidemiology] | 19233 |
| 26 | Mental health*·ti,ab· | 214636 |
| 27 | Psychiatr*·ti,ab· | 280241 |
| 28 | Mental illness*·ti,ab· | 39952 |
| 29 | Mental disorder*·ti,ab· | 49421 |
| 30 | Psychiatric illness*·ti,ab· | 10239 |
| 31 | Anxiety·ti,ab· | 260916 |
| 32 | Depression·ti,ab· | 417536 |
| 33 | ADHD·ti,ab· | 31450 |
| 34 | Autism·ti,ab· | 60105 |
| 35 | Autistic·ti,ab· | 16462 |
| 36 | Intellectual disabilit*·ti,ab· | 24458 |
| 37 | Attention Deficit Hyperactivity Disorder*·ti,ab· | 31093 |
| 38 | Psychiatric disorder*·ti,ab· | 51192 |
| 39 | Bipolar·ti,ab· | 73699 |
| 40 | PTSD·ti,ab· | 32491 |
| 41 | Post-traumatic stress disorder·ti,ab· | 15675 |
| 42 | Exp legislation/ | 1673 |
| 43 | Exp health policy/ | 133370 |
| 44 | Governance·ti,ab· | 19883 |
| 45 | Legislation·ti,ab· | 38599 |
| 46 | Regulations·ti,ab· | 57788 |
| 47 | Policy·ti,ab· | 248634 |
| 48 | Framework*·ti,ab· | 406679 |
| 49 | Reporting·ti,ab· | 265905 |
| 50 | (Legal approach or law or laws) ·ti,ab· | 133886 |
| 51 | Exp Informed Consent/ | 43675 |
| 52 | *Ethics, Research/ | 4577 |
| 53 | Parental Consent/ | 3356 |
| 54 | *Ethics Committees, Research/ | 2747 |
| 55 | *Research Subjects/lj | 204 |
| 56 | Consent·ti,ab· | 77959 |
| 57 | Assent·ti,ab· | 1228 |
| 58 | Ethics·ti,ab· | 73732 |
| 59 | Autonomy·ti,ab· | 37909 |
| 60 | Exp Africa/ | 327039 |
| 61 | ((Africa* or (Mali or Malis or Malian) or (Guinea* not “papua new guinea*”) or (Algeria or Angola or Benin or Botswana or “Burkina Faso” or Burundi or “Cabo Verde” or “Cape Verde” or Cameroon or “Central African Republic” or Chad or Comoros or Congo or “Cote d’Ivoire” or “Ivory Coast” or Djibouti or Egypt or “Equatorial Guinea” or Eritrea or Eswatini or Ethiopia or Gabon or Gambia or Ghana or “Guinea-Bissau” or Kenya or Lesotho or Liberia or Libya or Madagascar or Malawi or Mauritania or Mauritius or Morocco or Mozambique or Namibia or Niger or Nigeria or Rwanda or “Sao Tome” or Principe or Senegal or Seychelles or “Sierra Leone” or Somalia or “South Africa” or Sudan or Tanzania or Togo or Tunisia or Uganda or Zambia or Zimbabwe) or (((Algerian* or Angolan* or Beninois* or Batswana* or Burkinabe* or Cabo Verdean* or Cameroonian* or Central African* or Chadian* or Congolese* or Ivorian* or Djiboutian* or Egyptian* or Equatorial Guinean* or Eritrean* or Swazi* or Ethiopian* or Gabonese* or Gambian* or Ghanaian* or Guinean* or Bissau-Guinean* or Kenyan* or Mosotho* or Basotho* or Liberian* or Libyan* or Malawian* or Mauritanian* or Moroccan* or Mozambican* or Namibian* or Nigerien* or Nigerian* or Rwandan* or Rwandese* or Sao Tome) and Principe*) or Senegalese* or Sierra Leonean* or Creole* or Somalian* or South African* or South Sudanese* or Sudanese* or Tanzanian* or Togolese* or Tunisian* or Ugandan* or Zambian* or Zimbabwean*) or Comoro* or Upper Volta or Burundi* or French Somaliland or Gold Coast or Jamahiriya or Mayotte or Mocambique or Reunion or Basutoland or Malagasy Republic or Nyasaland or Ruanda Urundi or Rhodesia or Zaire or (“Western Sahara” or “Central Africa” or “Central African” or “West Africa” or “West African” or “Western Africa” or “Western African” or “East Africa” or “East African” or “Eastern Africa” or “Eastern African” or “North Africa” or “North African” or “Northern Africa” or “Northern African” or “South African” or “Southern Africa” or “Southern African” or “sub-sahara*” or “sub sahara*” or “subsahara*”)) not (“African American” or “black British”)) ·ti,ab· | 568844 |
| 62 | 1 or 2 or 3 or 4 or 5 or 6 or 7 or 8 or 9 or 10 or 11 or 12 or 13 or 14 or 15 or 16 or 17 or 18 | 4514766 |
| 63 | 19 or 20 or 21 or 22 or 23 or 24 or 25 or 26 or 27 or 28 or 29 or 30 or 31 or 32 or 33 or 34 or 35 or 36 or 37 or 38 or 39 or 40 or 41 | 2115750 |
| 64 | 42 or 43 or 44 or 45 or 46 or 47 or 48 or 49 or 50 or 51 or 52 or 53 or 54 or 55 or 56 or 57 or 58 or 59 | 1334357 |
| 65 | 60 or 61 | 638040 |
| 66 | 62 and 63 and 64 and 65 | 1525 |

| **Embase** | |
| --- | --- |
| **#** | **Searches** |
| 1 | exp Child/ |
| 2 | exp adolescent/ |
| 3 | exp Pediatrics/ |
| 4 | infant*·ti,ab· |
| 5 | toddler*·ti,ab· |
| 6 | child·ti,ab· |
| 7 | children·ti,ab· |
| 8 | teen*·ti,ab· |
| 9 | youth*·ti,ab· |
| 10 | youngster*·ti,ab· |
| 11 | adolescen*·ti,ab· |
| 12 | pre?school*·ti,ab· |
| 13 | kindergarten*·ti,ab· |
| 14 | school*·ti,ab· |
| 15 | juvenile*·ti,ab· |
| 16 | p?ediatric*·ti,ab· |
| 17 | (young adj2 (adult* or person* or individual* or people* or population* or man or men or wom#n)) ·ti,ab· |
| 18 | (secondary adj2 (school* or education)) ·ti,ab· |
| 19 | Mental Health/ |
| 20 | exp Mental Disorders/ |
| 21 | exp Psychiatry/ |
| 22 | exp Child Psychiatry/ |
| 23 | *Mental Health Services/ |
| 24 | *Intellectual Disability/ |
| 25 | *Depression/di, ep [Diagnosis, Epidemiology] |
| 26 | mental health*·ti,ab· |
| 27 | psychiatr*·ti,ab· |
| 28 | mental illness*·ti,ab· |
| 29 | mental disorder*·ti,ab· |
| 30 | psychiatric illness*·ti,ab· |
| 31 | anxiety·ti,ab· |
| 32 | depression·ti,ab· |
| 33 | ADHD·ti,ab· |
| 34 | autism·ti,ab· |
| 35 | autistic·ti,ab· |
| 36 | intellectual disabilit*·ti,ab· |
| 37 | Attention Deficit Hyperactivity Disorder*·ti,ab· |
| 38 | psychiatric disorder*·ti,ab· |
| 39 | bipolar·ti,ab· |
| 40 | PTSD·ti,ab· |
| 41 | post-traumatic stress disorder·ti,ab· |
| 42 | exp Legislation/ |
| 43 | exp Health Policy/ |
| 44 | governance·ti,ab· |
| 45 | legislation·ti,ab· |
| 46 | regulations·ti,ab· |
| 47 | policy·ti,ab· |
| 48 | framework*·ti,ab· |
| 49 | reporting·ti,ab· |
| 50 | (legal approach or law or laws) ·ti,ab· |
| 51 | exp Informed Consent/ |
| 52 | *Ethics, Research/ |
| 53 | Parental Consent/ |
| 54 | *Ethics Committees, Research/ |
| 55 | consent·ti,ab· |
| 56 | assent·ti,ab· |
| 57 | ethics·ti,ab· |
| 58 | autonomy·ti,ab· |
| 59 | exp Africa/ |
| 60 | ((Africa* or (Mali or Malis or Malian) or (Guinea* not "papua new guinea*") or (Algeria or Angola or Benin or Botswana or "Burkina Faso" or Burundi or "Cabo Verde" or "Cape Verde" or Cameroon or "Central African Republic" or Chad or Comoros or Congo or "Cote d'Ivoire" or "Ivory Coast" or Djibouti or Egypt or "Equatorial Guinea" or Eritrea or Eswatini or Ethiopia or Gabon or Gambia or Ghana or "Guinea-Bissau" or Kenya or Lesotho or Liberia or Libya or Madagascar or Malawi or Mauritania or Mauritius or Morocco or Mozambique or Namibia or Niger or Nigeria or Rwanda or "Sao Tome" or Principe or Senegal or Seychelles or "Sierra Leone" or Somalia or "South Africa" or Sudan or Tanzania or Togo or Tunisia or Uganda or Zambia or Zimbabwe) or (((Algerian* or Angolan* or Beninois* or Batswana* or Burkinabe* or Cabo Verdean* or Cameroonian* or Central African* or Chadian* or Congolese* or Ivorian* or Djiboutian* or Egyptian* or Equatorial Guinean* or Eritrean* or Swazi* or Ethiopian* or Gabonese* or Gambian* or Ghanaian* or Guinean* or Bissau-Guinean* or Kenyan* or Mosotho* or Basotho* or Liberian* or Libyan* or Malawian* or Mauritanian* or Moroccan* or Mozambican* or Namibian* or Nigerien* or Nigerian* or Rwandan* or Rwandese* or Sao Tome) and Principe*) or Senegalese* or Sierra Leonean* or Creole* or Somalian* or South African* or South Sudanese* or Sudanese* or Tanzanian* or Togolese* or Tunisian* or Ugandan* or Zambian* or Zimbabwean*) or Comoro* or Upper Volta or Burundi* or French Somaliland or Gold Coast or Jamahiriya or Mayotte or Mocambique or Reunion or Basutoland or Malagasy Republic or Nyasaland or Ruanda Urundi or Rhodesia or Zaire or ("Western Sahara" or "Central Africa" or "Central African" or "West Africa" or "West African" or "Western Africa" or "Western African" or "East Africa" or "East African" or "Eastern Africa" or "Eastern African" or "North Africa" or "North African" or "Northern Africa" or "Northern African" or "South African" or "Southern Africa" or "Southern African" or "sub-sahara*" or "sub sahara*" or subsahara*)) not ("African American" or "black british"))·ti,ab· |
| 61 | 1 or 2 or 3 or 4 or 5 or 6 or 7 or 8 or 9 or 10 or 11 or 12 or 13 or 14 or 15 or 16 or 17 or 18 |
| 62 | 19 or 20 or 21 or 22 or 23 or 24 or 25 or 26 or 27 or 28 or 29 or 30 or 31 or 32 or 33 or 34 or 35 or 36 or 37 or 38 or 39 or 40 or 41 |
| 63 | 59 or 60 |
| 64 | 42 or 43 or 44 or 45 or 46 or 47 or 48 or 49 or 50 or 51 or 52 or 53 or 54 or 55 or 56 or 57 or 58 |
| 65 | 61 and 62 and 63 and 64 |
| 66 | limit 65 to (embryo or infant or child or preschool child <1 to 6 years> or school child <7 to 12 years> or adolescent <13 to 17 years>) |

| **APA PsychInfo** | |
| --- | --- |
| # | **Searches** |
| 1 | exp child/ |
| 2 | exp adolescent/ |
| 3 | exp Pediatrics/ |
| 4 | infant*·ti,ab· |
| 5 | toddler*·ti,ab· |
| 6 | child·ti,ab· |
| 7 | children·ti,ab· |
| 8 | teen*·ti,ab· |
| 9 | youth*·ti,ab· |
| 10 | youngster*·ti,ab· |
| 11 | adolescen*·ti,ab· |
| 12 | pre?school*·ti,ab· |
| 13 | kindergarten*·ti,ab· |
| 14 | school*·ti,ab· |
| 15 | juvenile*·ti,ab· |
| 16 | p?ediatric*·ti,ab· |
| 17 | (young adj2 (adult* or person* or individual* or people* or population* or man or men or wom#n)) ·ti,ab· |
| 18 | (secondary adj2 (school* or education)) ·ti,ab· |
| 19 | Mental Health/ |
| 20 | exp Mental Disorders/ |
| 21 | exp Psychiatry/ |
| 22 | exp Child Psychiatry/ |
| 23 | *Mental Health Services/ |
| 24 | *Intellectual Disability/ |
| 25 | *Depression/di, ep [Diagnosis, Epidemiology] |
| 26 | mental health*·ti,ab· |
| 27 | psychiatr*·ti,ab· |
| 28 | mental illness*·ti,ab· |
| 29 | mental disorder*·ti,ab· |
| 30 | psychiatric illness*·ti,ab· |
| 31 | anxiety·ti,ab· |
| 32 | depression·ti,ab· |
| 33 | ADHD·ti,ab· |
| 34 | autism·ti,ab· |
| 35 | autistic·ti,ab· |
| 36 | intellectual disabilit*·ti,ab· |
| 37 | Attention Deficit Hyperactivity Disorder*·ti,ab· |
| 38 | psychiatric disorder*·ti,ab· |
| 39 | bipolar·ti,ab· |
| 40 | PTSD·ti,ab· |
| 41 | post-traumatic stress disorder·ti,ab· |
| 42 | exp Legislation/ |
| 43 | exp Health Policy/ |
| 44 | governance·ti,ab· |
| 45 | legislation·ti,ab· |
| 46 | regulations·ti,ab· |
| 47 | policy·ti,ab· |
| 48 | framework*·ti,ab· |
| 49 | reporting·ti,ab· |
| 50 | (legal approach or law or laws) ·ti,ab· |
| 51 | exp Informed Consent/ |
| 52 | *Ethics, Research/ |
| 53 | Parental Consent/ |
| 54 | *Ethics Committees, Research/ |
| 55 | consent·ti,ab· |
| 56 | assent·ti,ab· |
| 57 | ethics·ti,ab· |
| 58 | autonomy·ti,ab· |
| 59 | exp Africa/ |
| 60 | ((Africa* or (Mali or Malis or Malian) or (Guinea* not "papua new guinea*") or (Algeria or Angola or Benin or Botswana or "Burkina Faso" or Burundi or "Cabo Verde" or "Cape Verde" or Cameroon or "Central African Republic" or Chad or Comoros or Congo or "Cote d'Ivoire" or "Ivory Coast" or Djibouti or Egypt or "Equatorial Guinea" or Eritrea or Eswatini or Ethiopia or Gabon or Gambia or Ghana or "Guinea-Bissau" or Kenya or Lesotho or Liberia or Libya or Madagascar or Malawi or Mauritania or Mauritius or Morocco or Mozambique or Namibia or Niger or Nigeria or Rwanda or "Sao Tome" or Principe or Senegal or Seychelles or "Sierra Leone" or Somalia or "South Africa" or Sudan or Tanzania or Togo or Tunisia or Uganda or Zambia or Zimbabwe) or (((Algerian* or Angolan* or Beninois* or Batswana* or Burkinabe* or Cabo Verdean* or Cameroonian* or Central African* or Chadian* or Congolese* or Ivorian* or Djiboutian* or Egyptian* or Equatorial Guinean* or Eritrean* or Swazi* or Ethiopian* or Gabonese* or Gambian* or Ghanaian* or Guinean* or Bissau-Guinean* or Kenyan* or Mosotho* or Basotho* or Liberian* or Libyan* or Malawian* or Mauritanian* or Moroccan* or Mozambican* or Namibian* or Nigerien* or Nigerian* or Rwandan* or Rwandese* or Sao Tome) and Principe*) or Senegalese* or Sierra Leonean* or Creole* or Somalian* or South African* or South Sudanese* or Sudanese* or Tanzanian* or Togolese* or Tunisian* or Ugandan* or Zambian* or Zimbabwean*) or Comoro* or Upper Volta or Burundi* or French Somaliland or Gold Coast or Jamahiriya or Mayotte or Mocambique or Reunion or Basutoland or Malagasy Republic or Nyasaland or Ruanda Urundi or Rhodesia or Zaire or ("Western Sahara" or "Central Africa" or "Central African" or "West Africa" or "West African" or "Western Africa" or "Western African" or "East Africa" or "East African" or "Eastern Africa" or "Eastern African" or "North Africa" or "North African" or "Northern Africa" or "Northern African" or "South African" or "Southern Africa" or "Southern African" or "sub-sahara*" or "sub sahara*" or subsahara*)) not ("African American" or "black british"))·ti,ab· |
| 61 | 1 or 2 or 3 or 4 or 5 or 6 or 7 or 8 or 9 or 10 or 11 or 12 or 13 or 14 or 15 or 16 or 17 or 18 |
| 62 | 19 or 20 or 21 or 22 or 23 or 24 or 25 or 26 or 27 or 28 or 29 or 30 or 31 or 32 or 33 or 34 or 35 or 36 or 37 or 38 or 39 or 40 or 41 |
| 63 | 59 or 60 |
| 64 | 42 or 43 or 44 or 45 or 46 or 47 or 48 or 49 or 50 or 51 or 52 or 53 or 54 or 55 or 56 or 57 or 58 |
| 65 | 61 and 62 and 63 and 64 |

| **Global Health** | |
| --- | --- |
| **#** | **Searches** |
| 1 | exp child/ |
| 2 | exp adolescent/ |
| 3 | exp Pediatrics/ |
| 4 | infant*·ti,ab· |
| 5 | toddler*·ti,ab· |
| 6 | child·ti,ab· |
| 7 | children·ti,ab· |
| 8 | teen*·ti,ab· |
| 9 | youth*·ti,ab· |
| 10 | youngster*·ti,ab· |
| 11 | adolescen*·ti,ab· |
| 12 | pre?school*·ti,ab· |
| 13 | kindergarten*·ti,ab· |
| 14 | school*·ti,ab· |
| 15 | juvenile*·ti,ab· |
| 16 | p?ediatric*·ti,ab· |
| 17 | (young adj2 (adult* or person* or individual* or people* or population* or man or men or wom#n)) ·ti,ab· |
| 18 | (secondary adj2 (school* or education)) ·ti,ab· |
| 19 | Mental Health/ |
| 20 | exp Mental Disorders/ |
| 21 | exp Psychiatry/ |
| 22 | exp Child Psychiatry/ |
| 23 | *Mental Health Services/ |
| 24 | *Intellectual Disability/ |
| 25 | *Depression/di, ep [Diagnosis, Epidemiology] |
| 26 | mental health*·ti,ab· |
| 27 | psychiatr*·ti,ab· |
| 28 | mental illness*·ti,ab· |
| 29 | mental disorder*·ti,ab· |
| 30 | psychiatric illness*·ti,ab· |
| 31 | anxiety·ti,ab· |
| 32 | depression·ti,ab· |
| 33 | ADHD·ti,ab· |
| 34 | autism·ti,ab· |
| 35 | autistic·ti,ab· |
| 36 | intellectual disabilit*·ti,ab· |
| 37 | Attention Deficit Hyperactivity Disorder*·ti,ab· |
| 38 | psychiatric disorder*·ti,ab· |
| 39 | bipolar·ti,ab· |
| 40 | PTSD·ti,ab· |
| 41 | post-traumatic stress disorder·ti,ab· |
| 42 | exp Legislation/ |
| 43 | exp Health Policy/ |
| 44 | governance·ti,ab· |
| 45 | legislation·ti,ab· |
| 46 | regulations·ti,ab· |
| 47 | policy·ti,ab· |
| 48 | framework*·ti,ab· |
| 49 | reporting·ti,ab· |
| 50 | (legal approach or law or laws) ·ti,ab· |
| 51 | exp Informed Consent/ |
| 52 | *Ethics, Research/ |
| 53 | Parental Consent/ |
| 54 | *Ethics Committees, Research/ |
| 55 | consent·ti,ab· |
| 56 | assent·ti,ab· |
| 57 | ethics·ti,ab· |
| 58 | autonomy·ti,ab· |
| 59 | exp Africa/ |
| 60 | ((Africa* or (Mali or Malis or Malian) or (Guinea* not "papua new guinea*") or (Algeria or Angola or Benin or Botswana or "Burkina Faso" or Burundi or "Cabo Verde" or "Cape Verde" or Cameroon or "Central African Republic" or Chad or Comoros or Congo or "Cote d'Ivoire" or "Ivory Coast" or Djibouti or Egypt or "Equatorial Guinea" or Eritrea or Eswatini or Ethiopia or Gabon or Gambia or Ghana or "Guinea-Bissau" or Kenya or Lesotho or Liberia or Libya or Madagascar or Malawi or Mauritania or Mauritius or Morocco or Mozambique or Namibia or Niger or Nigeria or Rwanda or "Sao Tome" or Principe or Senegal or Seychelles or "Sierra Leone" or Somalia or "South Africa" or Sudan or Tanzania or Togo or Tunisia or Uganda or Zambia or Zimbabwe) or (((Algerian* or Angolan* or Beninois* or Batswana* or Burkinabe* or Cabo Verdean* or Cameroonian* or Central African* or Chadian* or Congolese* or Ivorian* or Djiboutian* or Egyptian* or Equatorial Guinean* or Eritrean* or Swazi* or Ethiopian* or Gabonese* or Gambian* or Ghanaian* or Guinean* or Bissau-Guinean* or Kenyan* or Mosotho* or Basotho* or Liberian* or Libyan* or Malawian* or Mauritanian* or Moroccan* or Mozambican* or Namibian* or Nigerien* or Nigerian* or Rwandan* or Rwandese* or Sao Tome) and Principe*) or Senegalese* or Sierra Leonean* or Creole* or Somalian* or South African* or South Sudanese* or Sudanese* or Tanzanian* or Togolese* or Tunisian* or Ugandan* or Zambian* or Zimbabwean*) or Comoro* or Upper Volta or Burundi* or French Somaliland or Gold Coast or Jamahiriya or Mayotte or Mocambique or Reunion or Basutoland or Malagasy Republic or Nyasaland or Ruanda Urundi or Rhodesia or Zaire or ("Western Sahara" or "Central Africa" or "Central African" or "West Africa" or "West African" or "Western Africa" or "Western African" or "East Africa" or "East African" or "Eastern Africa" or "Eastern African" or "North Africa" or "North African" or "Northern Africa" or "Northern African" or "South African" or "Southern Africa" or "Southern African" or "sub-sahara*" or "sub sahara*" or subsahara*)) not ("African American" or "black british"))·ti,ab· |
| 61 | 1 or 2 or 3 or 4 or 5 or 6 or 7 or 8 or 9 or 10 or 11 or 12 or 13 or 14 or 15 or 16 or 17 or 18 |
| 62 | 19 or 20 or 21 or 22 or 23 or 24 or 25 or 26 or 27 or 28 or 29 or 30 or 31 or 32 or 33 or 34 or 35 or 36 or 37 or 38 or 39 or 40 or 41 |
| 63 | 59 or 60 |
| 64 | 42 or 43 or 44 or 45 or 46 or 47 or 48 or 49 or 50 or 51 or 52 or 53 or 54 or 55 or 56 or 57 or 58 |
| 65 | 61 and 62 and 63 and 64 |

# **Appendix 3. Quality appraisal domains and appraisal of included studies**

**Narrative**^5^

Q1 Is the generator of the narrative a credible or appropriate source?

Q2 Is the relationship between the text and its context explained? (where, when, who with, how)

Q3 Does the narrative present the events using a logical sequence so the reader or listener can understand how it unfolds?

Q4 Do you, as reader or listener of the narrative, arrive at similar conclusions to those drawn by the narrator?

Q5 Do the conclusions flow from the narrative account?

Q6 Do you consider this account to be a narrative?

**Opinion**^5^

Q1 Is the source of the opinion clearly identified?

Q2 Does the source of opinion have standing in the field of expertise?

Q3 Are the interests of the relevant population the central focus of the opinion?

Q4 Does the opinion demonstrate a logically defended argument to support the conclusions drawn?

Q5 Is there reference to the extant literature?

Q6 Is any incongruence with the literature/sources logically defended?

**Case Series**^6^

Q1 Were there clear criteria for inclusion in the case series?

Q2 Was the condition measured in a standard, reliable way for all participants included in the case series?

Q3 Were valid methods used for identification of the condition for all participants included in the case series?

Q4 Did the case series have consecutive inclusion of participants?

Q5 Did the case series have complete inclusion of participants?

Q6 Was there clear reporting of the demographics of the participants in the study?

Q7 Was there clear reporting of clinical information of the participants?

Q8 Were the outcomes or follow up results of cases clearly reported?

Q9 Was there clear reporting of the presenting site(s)/clinic(s) demographic information?

Q10 Was statistical analysis appropriate?

**Qualitative**^7^

Q1 Is there congruity between the stated philosophical perspective and the research methodology?

Q2 Is there congruity between the research methodology and the research question or objectives?

Q3 Is there congruity between the research methodology and the methods used to collect data?

Q4 Is there congruity between the research methodology and the representation and analysis of data?

Q5 Is there congruity between the research methodology and the interpretation of results?

Q6 Is there a statement locating the researcher culturally or theoretically?

Q7 Is the influence of the researcher on their search, and vice-versa, addressed?

Q8 Are participant, and their voices, adequately represented?

Q9 Is the research ethical according to current criteria or, for recent studies, and is there evidence of ethical approval by an appropriate body?

Q10 Do the conclusions drawn in the research report flow from the analysis or interpretation, of the data?

**Systematic Reviews**^8^

Q1 Is the review question clearly and explicitly stated?

Q2 Were the inclusion criteria appropriate for the review question?

Q3 Was the search strategy appropriate?

Q4 Were the sources and resources used to search for studies adequate?

Q5 Were the criteria for appraising studies appropriate?

Q6 Was critical appraisal conducted by two or more reviewers independently?

Q7 Were there methods to minimize errors in data extraction?

Q8 Were the methods used to combine studies appropriate?

Q9 Was the likelihood of publication bias assessed?

Q10 Were recommendations for policy and/or practice supported by the reported data?

Q11 Were the specific directives for new research appropriate?

**Policy**^5^

Q1 Are the developers of the policy/ consensus guideline (and any allegiances/affiliations) clearly identified?

Q2 Do the developers of the policy/ consensus guideline have standing in the field of expertise?

Q3 Are appropriate stakeholders involved in developing the policy/guideline and do the conclusions drawn represent the views of their intended users?

Q4 Are biases due to competing interests acknowledged and responded to?

Q5 Are the processes of gathering and summarizing the evidence described?

Q6 Is any incongruence with the extant literature/evidence logically defended?

Q7 Are the methods used to develop recommendations described?

| **Textual Evidence: Narrative** | **Q1** | **Q2** | **Q3** | **Q4** | **Q5** | **Q6** |  |  |  |  |  |
| --- | --- | --- | --- | --- | --- | --- | --- | --- | --- | --- | --- |
| Mathai et al., 2022 | Yes | Yes | Yes | Yes | Yes | Yes |  |  |  |  |  |
| Madu et al., 2022 | Yes | Yes | Yes | Yes | Yes | Yes |  |  |  |  |  |
| Nienaber, 2013 | Yes | Yes | Yes | Yes | Yes | Yes |  |  |  |  |  |
| Gray et al., 2005 | Yes | Yes | Yes | Yes | Yes | Yes |  |  |  |  |  |
| Hertzog et al., 2021 | Yes | Yes | Yes | Yes | Yes | Yes |  |  |  |  |  |
| **Textual Evidence: Expert Opinion** | **Q1** | **Q2** | **Q3** | **Q4** | **Q5** | **Q6** |  |  |  |  |  |
| Parry et al., 1994 | Yes | Yes | Yes | Yes | Yes | Yes |  |  |  |  |  |
| Venturo-Conerly et al., 2022 | Yes | Yes | Yes | Yes | Yes | Yes |  |  |  |  |  |
| Bishop & Woolman, 2008 | Yes | Yes | No | Yes | Yes | Yes |  |  |  |  |  |
| **Case Series** | **Q1** | **Q2** | **Q3** | **Q4** | **Q5** | **Q6** | **Q7** | **Q8** | **Q9** | **Q10** |  |
| Woollett et al., 2017 | Yes | No | No | No | No | No | N/A | Yes | Yes | N/A |  |
| Betancourt et al., 2016 | Yes | No | Yes | Yes | Yes | Yes | Yes | Yes | No | N/A |  |
| **Qualitative** | **Q1** | **Q2** | **Q3** | **Q4** | **Q5** | **Q6** | **Q7** | **Q8** | **Q9** | **Q10** |  |
| Davids et al., 2019 | Yes | Yes | Yes | Yes | Yes | No | No | Yes | Yes | Yes |  |
| Kleintjes et al., 2010 | Unclear | Yes | Yes | Yes | Yes | No | No | Yes | Unclear | Yes |  |
| **Systematic Review** | **Q1** | **Q2** | **Q3** | **Q4** | **Q5** | **Q6** | **Q7** | **Q8** | **Q9** | **Q10** | **Q11** |
| Bakare et al., 2019 | Yes | Yes | Yes | Yes | N/A | N/A | Unclear | Unclear | No | Yes | Yes |
| **Policy** | **Q1** | **Q2** | **Q3** | **Q4** | **Q5** | **Q6** | **Q7** |  |  |  |  |
| Mokitimi et al., 2022 | Yes | Yes | Unclear | Yes | Yes | No | Yes |  |  |  |  |

# **Appendix 4. Study characteristics**

| ·· | **Provenance and Study Design** | **African Country Studied** | **Mental Health Conditions Discussed** |
| --- | --- | --- | --- |
| Bakare et al. (2019). | Journal article, scoping review | Nigeria | Autism Spectrum Disorder |
| Davids et al. (2019). | Journal article, qualitative | Ethiopia, Kenya, South Africa, Tunisia, Uganda, Zambia | General CAMH |
| Mathai et al. (2022). | Book chapter, NA | Kenya | General CAMH |
| Madu, E. N., & Osuji, H. (2022). | Book chapter, NA | Nigeria | General CAMH |
| Nienaber, A. (2013). | Journal article, narrative review | South Africa | General CAMH |
| Woollett et al. (2017). | Journal article, case study | South Africa | HIV-related mental health concerns |
| Betancourt et al. (2016). | Journal article, protocol development | Rwanda | HIV-related mental health concerns |
| Gray et al. (2005). | Journal article, literature review | South Africa | General CAMH |
| Parry, C. D. & Zwarenstein, M. (1994). | Journal article, letter to the editor | South Africa | General CAMH |
| Mokitimi et al. (2022). | Journal article, situational analysis | South Africa | General CAMH |
| Kleintjes et al. (2010). | Journal article, situational analysis | Ghana, South Africa, Uganda, Zambia | General CAMH |
| Venturo-Conerly et al. (2022). | Journal article, commentary | Kenya | Suicide Risk |
| Hertzog et al. (2021). | Journal article, commentary | South Africa | General CAMH |
| Bishop & Woolman (2008). | Legal document, NA | South Africa | General CAMH |

# **Appendix 5. Full-text exclusions**

| ·· | **Title** | **Reason for full-text exclusion** |
| --- | --- | --- |
| Anonymous (2013) | 10th International Congress on Adolescent Health | Not CAMH in Africa |
| Janse van Rensburg (2010) | Acute mental health care and South African mental health legislation: part 1- morbidity, treatment and outcome | Did not report findings specific to children |
| Sequeira et al. (2022) | Adolescent Health Series: The status of adolescent mental health research, practice and policy in sub-Saharan Africa: A narrative review | Did not report on CAMH research governance |
| Obasi et al. (2022) | Adolescent mental health research in Tanzania: a study protocol for a priority setting exercise and the development of an interinstitutional capacity strengthening programme | Did not report on CAMH research governance |
| Ichoku et al. (2013) | Africanizing the social determinants of health: embedded structural inequalities and current health outcomes in sub-Saharan Africa | Did not report findings specific to children |
| Divan et al. (2021) | Annual Research Review: Achieving universal health coverage for young children with autism spectrum disorder in low- and middle-income countries: a review of reviews | Did not report on CAMH research governance |
| Kleintjes (2019) | Barriers and opportunities to improving public health policy and service provision for children, adolescents and adults with intellectual and developmental disability: A South Africa case example | Not CAMH in Africa |
| Koelch et al. (2009) | ...because I am something special or "I think I will be something like a guinea pig": information and assent of legal minors in clinical trials--assessment of understanding, appreciation and reasoning | Not CAMH in Africa |
| Burgess et al. (2023) | Bringing an end to the silence: identifying priorities and solutions to addressing the mental health consequences of child marriage | Not CAMH in Africa |
| Compton (2020) | Challenges and future directions | Not CAMH in Africa |
| Robertson (2004) | Challenges for child and adolescent mental health service development in sub-Saharan Africa | Did not report on CAMH research governance |
| Flisher (2009) | Child and adolescent mental health in Africa: Some solutions | Did not report on CAMH research governance |
| Flisher et al. (2012) | Child and adolescent mental health in South Africa | Did not report on CAMH research governance |
| Mokitimi et al. (2018) | Child and adolescent mental health policy in South Africa: history, current policy development and implementation, and policy analysis | Did not report on CAMH research governance |
| Mokitimi et al. (2019) | Child and Adolescent Mental Health Services in South Africa-Senior Stakeholder Perceptions of Strengths, Weaknesses, Opportunities, and Threats in the Western Cape Province | Did not report on CAMH research governance |
| Bauta et al. (2022) | Child maltreatment and mental health in sub-Saharan Africa | Did not report on CAMH research governance |
| Kaliski (2001) | Dangerous legislation - Section 285A and the psychiatrist | Not CAMH in Africa |
| Singh et al. (2006) | Enrolling adolescents in research on HIV and other sensitive issues: lessons from South Africa | Not CAMH in Africa |
| Folayan et al. (2015) | Ethical Issues in Adolescents' Sexual and Reproductive Health Research in Nigeria | Not CAMH in Africa |
| Alonge et al. (2020) | Identifying pathways for large-scale implementation of a school-based mental health programme in the Eastern Mediterranean Region: a theory-driven approach | Did not report on CAMH research governance |
| Kleintjesi et al. (2020) | Improving the health of children and adults with intellectual disability in South Africa: legislative, policy and service development | Did not report on CAMH research governance |
| Berg (2011) | Infant mental health needs a model of service delivery | Did not report on CAMH research governance |
| Nyapadi (1995) | Legal aspects of medical research and the way forward | Not CAMH in Africa |
| Jordans et al. (2011) | Mental health interventions for children in adversity: pilot-testing a research strategy for treatment selection in low-income settings | Did not report on CAMH research governance |
| Ame et al. (2016) | Mental health law in Ghana: The rights of children with mental disorders | Did not report on CAMH research governance |
| Borba et al. (2016) | A mental health needs assessment of children and adolescents in post-conflict Liberia: results from a quantitative key-informant survey | Did not report on CAMH research governance |
| Kiima & Jenkins (2010) | Mental health policy in Kenya -an integrated approach to scaling up equitable care for poor populations | Did not report on CAMH research governance |
| Sharan et al. (2009) | Mental health research priorities in low- and middle-income countries of Africa, Asia, Latin America and the Caribbean | Did not report on CAMH research governance |
| Islam (1999) | Mental health services in the Seychelles | Not CAMH in Africa |
| Okasha (2019) | Psychosomatic medicine in Egypt and North Africa: Development, research, education and practice | Did not report on CAMH research governance |
| Dawes (2009) | The South African Children's Act | Not CAMH in Africa |
| Ssebunnya et al. (2011) | Stakeholders' perceptions of the main challenges facing Uganda's mental health care system: A qualitative analysis | Did not report on CAMH research governance |
| Mbwayo et al. (2022) | Strengthening System and Implementation Research Capacity for Child Mental Health and Family Well-being in Sub-Saharan Africa | Not CAMH in Africa |

#

# **Appendix 6. Descriptive themes definition and representative quote**

| **Descriptive Theme (number of studies with codes mapped onto this theme)** | **Definition** | **Representative Quote** |
| --- | --- | --- |
| Concerns over unfavourable existing legislation (n=4) | Barriers to conducting research due to existing legislation in place excluding target populations, finding the majority of research proposals unethical, and difficult processes to receive approval to start projects | In accordance with subsection 71(2), children and adolescents under the age of 18 years now require the consent of a parent or guardian to participate in mental health research. This requirement is in sharp contrast to the Choice on Termination of Pregnancy Act, according to which a minor female of any age may consent to the termination of her pregnancy; and to section 129 of the Children’s Act which, in fact, lowers the age of independent consent to medical treatment to 12 years of age (provided the child is of ‘sufficient maturity and has the mental capacity to understand the benefits, risks, social or other implications of the treatment or operation’).^2^ |
| Risks of conducting research (n=4) | Experiences of conducting child and adolescent mental health research in African countries where there may be very limited clinical service provision and laws may not consider this vulnerable population or how research can be carried out in the community | Without court intervention, most of the participants could not have participated in this research because parental consent was impossible to obtain. This case study argues for exceptions to the parental consent requirement, which excludes orphaned and vulnerable children and youth from research.^3^ |
| Call to action (n=7) | Lack of existing literature and no recognition for that which does exist leading to an urge for more research | [Challenges of CAMH are] dissemination of knowledge - a lot of research has been done, but we do not know about it because very few people publish. [We] keep citing that there is a paucity of research in an area while it is already there, we just do not have it published … The [emerging researchers] have the passion for CAMH and with the help of their mentors they are able to do more research that can lead to more evidence-based interventions.^4^ |
| Recommendations for future research (n=7) | What specifically researchers in this area want in terms of support | [We can transform CAMH] by networking as a continent. Drawing a robust training programme and monitor all trained personnel in their respective nations. Develop[ing] goals and targets for each nation on human resource development and research publications. EACH emerging research should have an attachment of mentors (senior scholars) for example, five years. These mentors should be from Africa and developed nations. |
| New directions for research (n=6) | Where are the greatest research gaps | Research is urgently needed into several ethical and legal issues surrounding children and adolescents’ consent to participation in mental health research. This includes research into the capacity to consent to research participation, in order to determine the extent to which consent is limited by certain mental disorders, as well as the clinical assessment of the capacity to consent.29 |

# **Appendix 7: References**

1. Page MJ, McKenzie JE, Bossuyt PM, Boutron I, Hoffmann TC, Mulrow CD, et al. The PRISMA 2020 statement: an updated guideline for reporting systematic reviews. BMJ [Internet]. 2021 Mar 29 [cited 2024 Jul 8];372. Available from: https://www.bmj.com/content/372/bmj.n71

2. Nienaber AG. Consent to research by mentally ill children and adolescents: The implications of Chapter 9 of the National Health Act. South African Journal of Psychiatry [Internet]. 2013 Mar 1 [cited 2024 May 11];19(1):5. Available from: https://sajp.org.za/index.php/sajp/article/view/386/372

3. Woollett N, Peter J, Cluver L, Brahmbhatt H. Enrolling HIV-positive adolescents in mental health research: A case study reflecting on legal and ethical complexities. South African Medical Journal [Internet]. 2017 Jul 28 [cited 2024 May 11];107(8):679–83. Available from: http://www.samj.org.za/index.php/samj/article/view/12020

4. Davids EL, Adams Tucker L, Wambua GN, Fewster DL, Schlebusch L, Karrim SBS, et al. Child and adolescent mental health in Africa: A qualitative analysis of the perspectives of emerging mental health clinicians and researchers using an online platform. J Child Adolesc Ment Health [Internet]. 2019 May 4 [cited 2024 May 11];31(2):93–107. Available from: https://www.tandfonline.com/doi/abs/10.2989/17280583.2019.1659145

5. McArthur A, Klugarova J, Yan H, Florescu S. Systematic reviews of text and opinion. In: Aromataris E, Lockwood C, Porritt K, Pilla B, Jordan Z, editors. JBI Manual for Evidence Synthesis. JBI; 2020.

6. Munn Z, Barker TH, Moola S, Tufanaru C, Stern C, McArthur A, et al. Methodological quality of case series studies: An introduction to the JBI critical appraisal tool. JBI Database System Rev Implement Rep [Internet]. 2019 [cited 2024 Jun 12]; Available from: https://journals.lww.com/jbisrir/fulltext/2020/10000/methodological_quality_of_case_series_studies__an.5.aspx

7. Lockwood C, Munn Z, Porritt K. Qualitative research synthesis: Methodological guidance for systematic reviewers utilizing meta-aggregation. Int J Evid Based Healthc [Internet]. 2015 Sep 1 [cited 2024 Jun 12];13(3):179–87. Available from: https://journals.lww.com/ijebh/fulltext/2015/09000/qualitative_research_synthesis__methodological.10.aspx

8. Aromataris E, Fernandez R, Godfrey CM, Holly C, Khalil H, Tungpunkom P. Summarizing systematic reviews: methodological development, conduct and reporting of an umbrella review approach review of reviews, systematic review, umbrella review, umbrella review methodology. Int J Evid Based Healthc [Internet]. 2015 [cited 2024 Jun 12]; Available from: https://journals.lww.com/ijebh
